# Microeukaryotic gut parasites in wastewater treatment plants: diversity, activity, and removal

**DOI:** 10.1186/s40168-022-01225-y

**Published:** 2022-02-09

**Authors:** Jule Freudenthal, Feng Ju, Helmut Bürgmann, Kenneth Dumack

**Affiliations:** 1 Terrestrial Ecology, Institute of Zoology, University of Cologne, Zülpicher Str. 47b, 50674 Köln, Germany; 2 Key Laboratory of Coastal Environment and Resources of Zhejiang Province, School of Engineering, Westlake University, Hangzhou, 310024 China; 3 Institute of Advanced Technology, Westlake Institute for Advanced Study, Hangzhou, 310024 China; 4 Eawag, Swiss Federal Institute of Aquatic Science and Technology, 6047 Kastanienbaum, Switzerland

**Keywords:** Protists, Parasite removal, Water treatment, Food web, Metatranscriptomics, Metagenomics

## Abstract

**Background:**

During wastewater treatment, the wastewater microbiome facilitates the degradation of organic matter, reduction of nutrients, and removal of gut parasites. While the latter function is essential to minimize public health risks, the range of parasites involved and how they are removed is still poorly understood.

**Results:**

Using shotgun metagenomic (DNA) and metatranscriptomic (RNA) sequencing data from ten wastewater treatment plants in Switzerland, we were able to assess the entire wastewater microbiome, including the often neglected microeukaryotes (protists). In the latter group, we found a surprising richness and relative abundance of active parasites, particularly in the inflow. Using network analysis, we tracked these taxa across the various treatment compartments and linked their removal to trophic interactions.

**Conclusions:**

Our results indicate that the combination of DNA and RNA data is essential for assessing the full spectrum of taxa present in wastewater. In particular, we shed light on an important but poorly understood function of wastewater treatment – parasite removal.

Video Abstract

**Supplementary Information:**

The online version contains supplementary material available at 10.1186/s40168-022-01225-y.

## Background

The microbiome in wastewater treatment plants (WWTPs) includes not only prokaryotes but also eukaryotes: fungi, protists, and microscopic metazoans. Together, this wastewater community facilitates anaerobic denitrification and aerobic nitrification, as well as heterotrophic respiration and flocculation [1–5]. Specifically, the coupling of denitrification and nitrification reduces wastewater nitrogen, while aerobic and anaerobic heterotrophs help to degrade organic material [1, 4]. Microbial biomass growth leads to flocculation, enabling the separation of solids through sedimentation [2, 3].

Although the abovementioned functions are fulfilled by the concerted actions of the entire WWTP microbiome, the vast majority of WWTP surveys focus merely on the most numerous microbial entity in WWTPs – bacteria [6]. Protists, in particular, remain largely underappreciated, despite representing the majority of the eukaryotic fraction [7–9]. Although they are less numerous than bacteria, protists profoundly affect the community composition of their prokaryotic and eukaryotic prey [10–12]. In WWTPs, protists have been found to modulate the composition and biomass of the microbial community, thus affecting denitrification, nitrification, and flocculation, for instance by feeding on filamentous bacteria or loosely attached bacteria from flocs [2, 3, 13, 14].

In addition to their key role in regulating the WWTP microbial community, protists deserve more attention for another important reason: this microeukaryotic group includes many gut-associated taxa that are potentially harmful to humans and animals [12, 15]. The removal of these parasites, which include taxa such as *Giardia* and *Entamoeba*, is a key function of wastewater treatment [16–19]. However, little is known about the mechanisms involved, including the role of predation.

This knowledge gap is to a large degree due to the challenges involved in the taxonomic identification and enumeration of the main predators in wastewater – protists [1]. Apart from labor-intensive microscopy, primer-based metabarcoding is currently the most commonly used method to assess microbiomes in various environments, including sewage. However, this method is inevitably selective as there is no general primer that enables the assessment of all taxa present leading to contradictory results in protist assessments [20–23]. Another alternative is to use shotgun methods, which are primer-independent and thus suitable for assessing microbial communities in their entirety, including (parasitic) protists [24, 25]. Specifically, shotgun metagenomics (DNA-based) are used to determine microbial community composition and functional potential, while shotgun metatranscriptomics (RNA-based) provides a proxy for assessing microbial activity [26, 27]. So far, these promising methods have rarely been used to investigate microbial communities in sewage or WWTPs on a large scale, and if so, the data were not screened for protists.

The present study addresses this gap by analyzing a publicly available data set of shotgun metagenomic and metatranscriptomic data provided by Ju et al. [28], who sampled microbial communities in various treatment compartments of WWTPs across Switzerland. This data set allowed us to assess the WWTP microbial community as a whole, including protists, and without a primer bias. Our specific objective was to identify protist taxa that are potential gut parasites, track their abundance and activity patterns across the consecutive WWTP compartments (from inflow to effluent), and screen for putative predator-prey interactions that could explain parasite removal during wastewater treatment.

## Material and methods

We made use of the publicly available data sets from Ju et al. [28]. In brief, these authors sampled 12 WWTPs across Switzerland for DNA (shotgun metagenomics) and RNA (shotgun metatranscriptomics). At each facility, they sampled four compartments connected by continuous flow: sewage-inflow after screening and primary sedimentation (INF), denitrification bioreactor (DNF), nitrification bioreactor (NFC), and effluent after passing of the secondary clarifier (EFF). For details of the sampling process and metagenomic and metatranscriptomic sequencing, see Ju et al. [28]. As explained below, after initial data processing, we based our final analysis on data from 10 of the 12 WWTPs sampled.

### Data processing

We used Ju et al.’s [28] metagenomic data (DNA) to assess the WWTP community in terms of taxonomic composition, and their metatranscriptomic data (RNA) as a measure of metabolic and reproductive activity [26, 27]. We assessed the raw data via MG-RAST [29] and made use of the implemented MG-RAST prefiltering and ribosomal sequence calling. All statistical analyses and data visualizations described in the present paper, unless otherwise stated, were performed with the packages ggpubr v. 0.4.0 [30], rstatix v. 0.7.0 [31], SpiecEasi v. 1.1.0 [32], and vegan v. 2.5-7 [33] in R v. 3.6.2 [34]. All figures, except the networks, were produced using ggthemes v. 4.2.4 [35], ggplot2 v. 3.3.5 [36], and ggpubr v. 0.4.0 [30]. The networks were visualized using Cytoscape v. 3.8.0 [37].

To identify prokaryotic taxa (bacteria and archaea) in the WWTP samples, we searched for sequence similarities in the SILVA data base [38]. Similarly, to identify eukaryotic taxa (protists, fungi, and microscopic metazoa), we searched the PR^2^ data base [39]. Using BLASTN [40], we filtered the search results using an *e* value of 1e^-50^ and a similarity threshold of ≥ 80 %, keeping only the best hit. Given the limitations of the sequencing method (read length of ~ 150 bp per fragment, limited sequencing depth, and sequencing of random fragments), we binned sequences at genus level, to avoid overestimation of microbial diversity in the data set [28, 41, 42]. Singletons were removed and putative contaminants, such as sequences derived from macroscopic animals, higher plants (Streptophyta), and chloroplasts, were excluded. For convenience, in this paper, we refer to the assessed communities as “microbial communities,” although they also include microscopic fauna (gastrotrichs, nematodes, rotifers, and tardigrades).

Considering that WWTP microbial communities are affected by location-specific environmental and operational factors, and therefore cannot necessarily be treated as biological replicates, we screened the data for potential outliers [1]. To this end, we compared the microbial communities of the different WWTP locations by exploring multivariate dispersion (visualized by non-metric multidimensional scaling, NMDS, function metaMDS, package vegan, Supplementary Fig. 1 A) and beta diversity (function vegdist, package vegan, Supplementary Fig. 1 B). Bray-Curtis dissimilarity was calculated from the relative abundance data, i.e., the number of reads of each taxon was divided by the total number of reads of the respective sample. Significant differences in beta diversity were identified using unpaired two-sample Wilcoxon tests (function stat_compare_means, package ggpubr). Based on these results, WWTP location “FD” [28] was removed from subsequent analyses due to clear differences in beta diversity (Supplementary Fig. 1). In addition, location “BE” was removed because its design prevented the sampling of its denitrification bioreactor. Consequentially, our further analysis focused on 10 out of the originally 12 WWTPs sampled by Ju et al. [28].

For these ten locations, sequence data were subsampled (rarefied) to guarantee a similar sampling depth of ribosomal (marker) gene sequences across the entire range of DNA and RNA data, respectively. Prior to rarefaction, one RNA sample and two DNA samples were removed from the data set because of exceptionally low sequencing depth in ribosomal genes. Accordingly, the data were rarefied to a depth of 13,359 DNA and 13,812 RNA marker gene sequences per sample.

Furthermore, we evaluated the variation caused by sample processing, i.e., sequencing. When Ju et al. [28] sampled the WWTPs for the database used in our study, they collected one sample per compartment, except for WWTP location “ZR”, where an additional two replicates in the inflow compartment were subjected to sequencing to assess the technical variation. We evaluated this variation based on an NMDS plot made with relative abundance data transformed by Bray-Curtis dissimilarity (metaMDS function, package vegan; Supplementary Fig. 2). This analysis showed that variation caused by sequencing was low. For the remainder of our analyses, we kept only one of the three replicates mentioned above, to ensure comparability with the single samples taken from the other compartments at the different WWTP locations.

Rarefaction curves were calculated from count data using the function rarecurve (package vegan). With a total richness of 1947 and 1887 operational taxonomical units (OTUs) identified in the rDNA and rRNA data respectively, rarefaction curves showed sufficient saturation in sequencing (Supplementary Fig. 3). An overview of the number of reads and OTUs of prokaryotes, protists, fungi, and microscopic metazoans is provided in Supplementary Table 1.

Finally, functional traits were assigned to the taxa identified, using published reference databases [43–48]. Based on these trait databases, we classified the following taxa as parasites: (a) all protist genera associated with human and animal gut and/or feces and (b) all prokaryote, fungal, and microscopic-metazoan genera that include potentially pathogenic species to humans and animals. The poorly investigated and difficult to detect *Rosculus* and *Guttulinopsis*, two protistan taxa that are primarily known from feces of livestock for which evidence of a complete gut passage is yet missing, are here also considered as parasites [49]. An overview of the parasitic genera thus identified is given in Supplementary Table 2.

### Area plots, line plots, and box plots

To analyze microbial community changes across wastewater treatment compartments, we computed area plots of the 11 most abundant prokaryotic and eukaryotic orders, including both free-living and parasitic taxa. Differences between the treatment compartments were tested both in terms of community composition (rDNA) and activity (rRNA), using Permutational Multivariate Analysis of Variance (PERMANOVA, adonis function, package vegan). Changes in the total number of ribosomal sequences over time, i.e., across the consecutive compartments, were analyzed based on qPCR analysis for DNA sequencing and spiked internal standards for RNA sequencing (RIS) (Supplementary Fig. 4). Total abundances of eukaryotic ribosomal sequences were estimated based on the relative proportion of shotgun data in relation to the total abundances of qPCR prokaryotic ribosomal sequences. Differences between the total abundance in the inflow versus denitrification bioreactor, the denitrification versus nitrification bioreactor, and the nitrification bioreactor versus the outflow were tested using sign test (function sign_test, package rstatix, Supplementary Table 3). Additionally, the relative abundance of selected parasitic protist taxa over time were visualized in line plots and tested for significant differences using sign test (function sign_test, package rstatix), comparing their relative abundance in the inflow versus denitrification bioreactor, and the nitrification bioreactor versus the outflow (Supplementary Table 4). Finally, to evaluate differences between measurable presence and activity, we compared the relative abundance of rDNA versus rRNA reads. This was done for the most numerous orders within the community, across all compartments (Supplementary Table 5), as well as for the seven parasitic protist taxa mentioned above, focusing on the inflow compartment where they were most abundant (box plots). For the latter, differences between rDNA and rRNA relative abundance were determined by Sign test, not considering outliers (package rstatix).

**Fig. 1:**
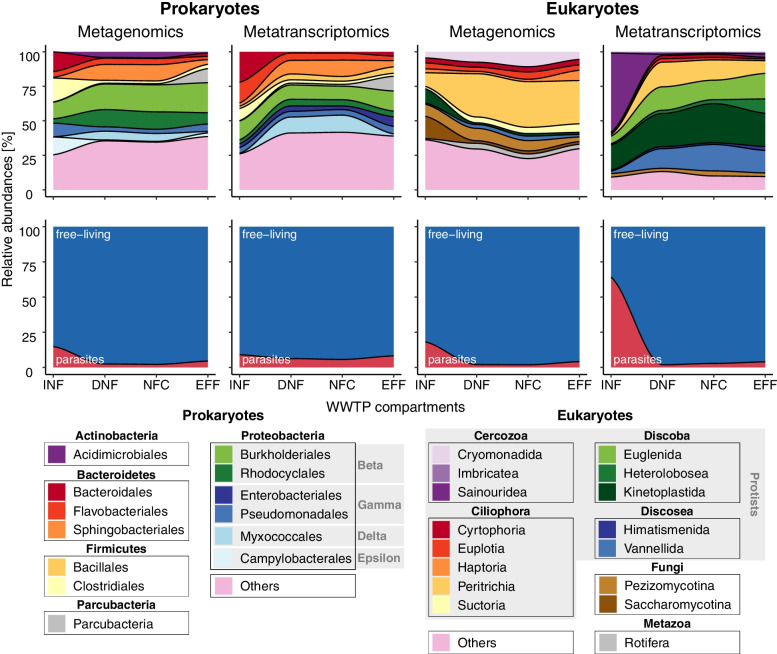
Changes in microbial community composition during wastewater treatment. Area plots showing microbial community composition in four consecutive compartments: INF inflow (sewage), DNF denitrification bioreactor, NFC nitrification bioreactor, and EFF effluent. Numbers in A represent the mean relative abundance (across *N*=10 WWTP locations) of the most abundant orders and, in B, the mean proportion of potential parasites versus free-living taxa. Numbers are shown separately for prokaryotic taxa (1st and 2nd column) and eukaryotic taxa (3rd and 4th column), comparing relative abundance based on rDNA reads (metagenomics) and rRNA reads (metatranscriptomics)

**Fig. 2:**
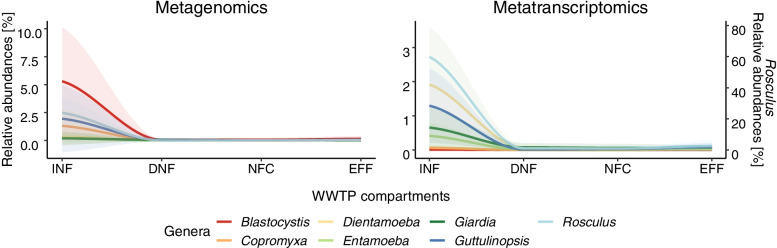
Removal of parasitic protists from wastewater. Line plots showing mean relative abundance of selected parasitic protist genera across the four WWTP compartments (INF inflow (sewage), DNF denitrification bioreactor, NFC nitrification bioreactor, EFF effluent). Numbers shown are mean relative protist abundances (across *N*=10 WWTP locations) of rDNA reads (metagenomics, left-hand side) and rRNA reads (metatranscriptomics, right-hand side). In the latter, abundance of the genus *Rosculus* is shown on a separate *Y*-axis because of its high number of rRNA reads. Standard deviations are indicated by the transparent areas (colors matching with individual lines)

**Fig. 3:**
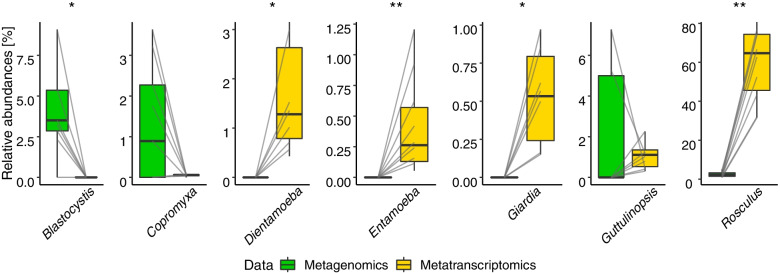
Detection of parasitic protists in wastewater: abundance versus activity. Boxplots showing, for selected taxa, the 25 % and 75 % percentiles and medians of the relative abundance of protist reads in metagenomic data (rDNA, green) and metatranscriptomic data (rRNA, yellow), in samples from the inflow compartment (at *N*=10 WWTP locations). Significant differences between the rDNA and rRNA abundances are indicated with asterisks (sign test, *p< 0.05, **p< 0.01, ***p< 0.001). The gray lines link the rDNA and rRNA sample pairs from the same location

### Network inference

Co-occurrence network analyses were performed to assess the complexity of correlations between free-living and parasitic taxa within the WWTP microbial community and draw inferences about the role of predation in parasite removal. Beforehand, we conducted two pre-processing steps to reduce indirect associations (spurious edges).

First, we reduced spurious edges caused by environmental factors. In network inference, it is a challenge to disentangle microbial associations reflecting ecological relationships–direct edges–from those induced by the environment – indirect edges [50–53]. To evaluate the influence of environmental factors on the WWTP microbial community, NMDS plots were computed for the WWTPs as a whole (Supplementary Fig. 5) as well as for the individual compartment types (Supplementary Fig. 6), for both rDNA and rRNA relative abundance data (function metaMDS, package vegan). Next, the environmental data measured by Ju et al. [28] (pH, dissolved oxygen, dissolved organic carbon, total nitrogen, total phosphorus, temperature, and hydraulic retention time) were fitted onto the ordinations using envfit (vegan). The resulting *p* values were corrected for multiple testing according to Benjamini & Hochberg [54]. Significant environmental vectors, scaled (multiplied) with their correlation value, were added to the NMDS plots. This analysis showed that environmental factors such as pH, total phosphorus (TP), and dissolved organic matter (DOC) correlated significantly with the diversity of the microbial community at the WWTP level (Supplementary Fig. 5), but not at the individual compartment level (with one exception; see Supplementary Fig. 6). Thus, spurious edges caused by environmental factors were minimized by conducting the network analysis at the compartment level.

Secondly, we reduced spurious edges caused by rare species. Since co-absence can yield artificially high correlation values that have no ecological meaning [50], we filtered the data for rare taxa. Thus, for each WWTP compartment type, we excluded taxa detected in fewer than seven samples (of *N*=10 samples across WWTP locations). Metagenomic and metatranscriptomic data were processed separately.

Following these two pre-processing steps, network analysis was conducted by combining two methods, i.e., Sparse Correlations for Compositional data (SparCC) and Sparse and Compositionally Robust Inference of Microbial Ecological Networks (SPIEC-EASI), as suggested by Chen et al. [55]. SparCC accounts for compositionality using a correlation measure derived from Aitchison’s variance of log-ratios [56], while SPIEC-EASI, in addition to accounting for compositionality, also reduces indirect edges by using sparse neighborhood or inverse covariance selection to infer correlations [32]. Since each method relies on different approaches to optimally filter noises and none performs across all data sets, we combined the two methods in an attempt to improve the prediction accuracy [55, 57, 58].

To combine SparCC and SPIEC-EASI methods, networks were first calculated with each approach separately. The same workflow was used for each WWTP compartment type and conducted separately for rDNA and rRNA data. For the Python (v. 2.7.18) based SparCC function non-normalized count data were used (package sparcc, v. 0.1.0, Friedman & Alm, 2012). Significant correlations at False Discovery Rate 0.05 were obtained by 100 permutations of randomly shuffled data (function MakeBootstraps, package sparcc), subjected to network inference. SPIEC-EASI networks (function spiec.easi, package SpiecEasi), based on non-normalized count data as well, were calculated with the sparse Meinshausen-Buhlmann’s neighborhood selection (mb) method [32]. The default scaling factor determining the minimum sparsity (lambda.min.ratio) was lowered to 0.001 because of the density of the networks. In order to get closer to the target stability threshold (0.05), nlambda was set to 50. Finally, only the shared correlations of both network inference methods were retained and visualized in Cytoscape v. 3.8.0 [37] (Supplementary Fig. 7). The complexity of the networks was reduced for visualization [51]. To this end, the nodes were grouped at order level, with the edges indicating the number of genera with shown correlations. To further reduce the complexity of the graphs, only correlations with parasitic taxa are shown in this paper.

**Fig. 4:**
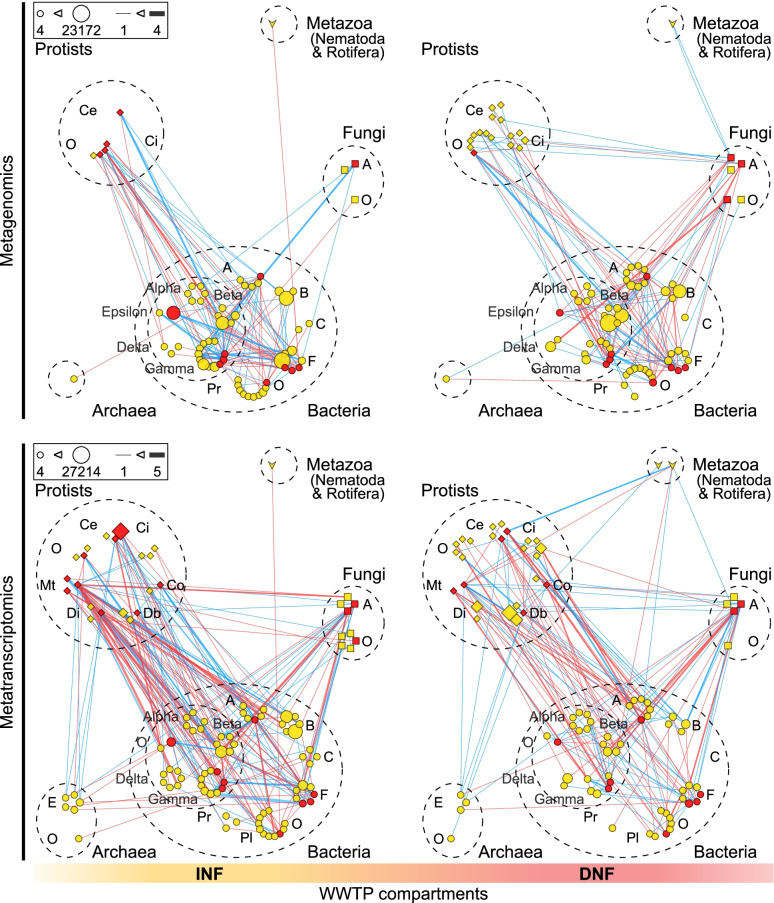
Co-occurrence networks of parasitic orders in the inflow and denitrification bioreactor. Networks showing correlations derived from co-occurrence network inferences for the inflow (INF) and denitrification bioreactor (DNF) at *N*=10 WWTP locations, based on metagenomic (first row) and metatranscriptomic (second row) data. Only associations that involve parasites are shown. Nodes represent genera grouped at the order level and trait level (red nodes: parasitic taxa; yellow nodes: free-living taxa), with node size proportional to the total number of reads for each order. Edges represent correlations between taxa (blue lines: positive correlations; red lines: negative correlations), with line thickness proportional to the number of genera per order involved. Abbreviations for archaea: E Euryarchaeota, O others. Abbreviations for bacteria: A Actinobacteria, B Bacteroidetes, C Chloroflexi, F Firmicutes, O Others, Pl Planctomycetes, Pr Proteobacteria, T Tenericutes, V Verrucomicrobia. Abbreviations for Proteobacteria: Alpha Alphaproteobacteria, Beta Betaproteobacteria, Gamma Gammaproteobacteria, Delta Deltaproteobacteria, O others. Abbreviations: Fungi: A Ascomycota, B Basidiomycota, O others. Abbreviations for protists: Ce Cercozoa (*including *Rosculus*), Ci Ciliophora, Co Conosa, Db Discoba, Di Discosea, Ms Mesomycetozoa, Mt Metamonada, O Others, S Stramenopiles, T Tubulinea

## Results

The taxonomic richness of microbial organisms associated with wastewater treatment was found to be high (Supplementary Table 1). We identified a total of 508,250 SSU rDNA sequences via metagenomics and 537,935 SSU rRNA sequences via metatranscriptomics and assigned these to 1947 and 1887 operational taxonomic units (OTUs), respectively. Prokaryotes constituted ~ 94.3 % of the rDNA reads, but only ~ 42.5 % of the rRNA reads. Conversely, protists were less dominant in the rDNA reads (~ 4.6 %) but represented as much as ~ 54.8 % of the rRNA reads. Fungi and microscopic metazoa represented only minor fractions, with slightly higher contributions to the rRNA reads (~ 1.9 % and ~ 0.9 %, respectively) than rDNA reads (~ 0.7 % and ~ 0.4 %, respectively).

In terms of community composition (rDNA, metagenomics), the prokaryotic community was dominated by the orders Burkholderiales, Rhodocyclales, and Sphingobacteriales (bacteria), and the eukaryotic community by the protist orders Peritrichia (Ciliophora) and Cryomonadida (Cercozoa), and the fungal order Pezizomycotina (Fig. 1). The highest activity (rRNA, metatranscriptomics) was found in the prokaryotic orders Burkholderiales, Flavobacteriales, and Sphingobacteriales (bacteria) and the protist orders Kinetoplastida (Discoba), Sainouridea (Cercozoa), and Euglenida (Discoba) (Fig. 1). Taxonomic composition differed significantly between the four WWTP compartments (PERMANOVA, rDNA: *R*^2^ = 0.59, *p* = 0.001; rRNA: *R*^2^ = 0.35, *p* = 0.001), reflecting community changes during the wastewater treatment process (Supplementary Fig. 5). Interestingly, across all compartments, the eukaryotic taxa showed pronounced differences between their relative abundance of rDNA (a measure of biomass) and relative abundance of rRNA (a measure of activity), while these differences were much less pronounced in the prokaryotic taxa (Fig. 1, Supplementary Table 5).

In terms of parasitic taxa, we found that the WWTP microbiome included a large and diverse number of parasitic eukaryotes (up to ~ 64 % of the eukaryote reads, of which the majority represented parasitic protists and less than 1% represented other parasitic eukaryotes) and a relatively smaller fraction of parasitic bacteria (up to ~ 15 % of the prokaryote reads). Effective removal of parasites over the course of wastewater treatment was indicated by their pronounced decrease from high abundance in the inflow (mostly raw sewage) to low abundance in the outflow (effluent), both in relative numbers (Fig. 1) and total numbers of ribosomal sequences (Supplementary Fig. 4).

Based on these observations, we investigated the progress of parasite removal during wastewater treatment in closer detail by comparing changes in relative abundance and activity across WWTP compartments, focusing on selected taxa of parasitic protists (Fig. 2). Here, when comparing the inflow (INF) to denitrification (DNF) compartments, significant decreases were found in the relative abundance of *Blastocystis* and *Rosculus*, and in the activity of all taxa except *Blastocystis* (Sign-Test, Supplementary Table 4). When comparing the nitrification (NFC) bioreactor to the outflow (EFF) compartment, significant increases were found in the activity of *Copromyxa* and *Rosculus* (Sign-Test, Supplementary Table 4).

Given these different changes in abundance versus activity, we contrasted the “detectability” of each of the protist taxa mentioned above, in terms of abundance (rDNA, metagenomics) versus activity (rRNA, metatranscriptomics), focusing on the samples from the inflow (Fig. 3). Intriguingly, this comparison showed that the gut parasites *Dientamoeba*, *Entamoeba*, *Giardia*, and *Rosculus* were hardly detectable in terms of abundance (rDNA) but yielded a high number of reads in terms of activity (rRNA). Conversely, the gut parasites *Blastocystis*, *Copromyxa*, and *Guttulinopsis* were hardly detectable in terms of activity (rRNA) but were clearly present in most rDNA samples. In other words, when present, the latter taxa showed low or no measurable activity.

To investigate whether parasite abundance and activity patterns across wastewater treatment can be explained by microbial community interactions, we conducted network analyses, looking specifically for associations between parasites and potential competitors, predators, and co-associated parasites. When comparing the networks of the communities in the inflow versus denitrification compartments, where most of the parasite removal took place, we observed a surprisingly high number of correlations between bacteria and eukaryotes, in addition to the commonly reported correlations within bacteria (Fig. 4; for other compartments, see Supplementary Fig. 7). Across the networks shown in Fig. 4, correlations within the bacteria accounted for ~ 47 % of all correlations, while correlations between bacteria and eukaryotes accounted for ~ 44 %. Of the latter correlations, 74 % involved protists. Particularly interesting are the correlations found for *Rosculus*, the main genus found in the Cercozoa (“Ce” in Fig. 4). This parasitic and bacterivorous protist was found to be highly active (up to ~ 84 % of the protist rRNA reads) in the inflow, with significantly lower readings in the denitrification bioreactor (only ~ 4 % of the protist reads, Fig. 2, Sign-Test, Supplementary Table 4). Network inference revealed that, in the inflow, *Rosculus* correlated exclusively with bacteria (indicating *Rosculus* feeding on bacteria) while, in the denitrification compartment, it had fewer correlations with bacteria but gained correlations with the rotifers *Adenita* and *Monostyla* (indicating *Rosculus* being preyed upon by rotifers). Other parasitic protist taxa followed the same general pattern of strongly decreasing numbers between the inflow and denitrification compartments (Fig. 2), correlating with bacteria, fungi, and other smaller protists in the inflow, and gaining correlations with predatory ciliates and rotifers in the denitrification bioreactor (Fig. 4).

Interestingly, the correlations discussed above emerged more strongly from the networks derived from activity data (rRNA, metatranscriptomics) than from the networks derived from abundance data (rDNA, metagenomics). As shown in Fig. 4, rDNA and rRNA networks showed distinct differences in density, with respectively 124 versus 192 edges (correlations) and 65 versus 104 nodes (taxa) in the inflow, and 135 versus 151 edges and 78 versus 85 nodes in the denitrification compartment. This greater density of rRNA networks was also observed in the other compartments (Supplementary Fig. 7). Moreover, the rRNA networks revealed associations between parasitic protists and their potential predators (rotifers, in particular) that were not detected in the rDNA networks.

## Discussion

Our study provides a comprehensive overview of the diversity of microorganisms in wastewater treatment plants (WWTPs), including not only bacteria but also fungi, protists, and microscopic metazoans. Expectedly, bacteria represented the most numerous fraction of the microbial community in terms of metagenomics [6]. However, in terms of metatranscriptomics (activity), the number of eukaryotic reads was higher than the prokaryotic reads. Surprisingly, within the eukaryotic fraction, the abundance and activity of protists were found to be much higher than of fungi. With ~ 55% of all rRNA reads, protists, including many potential parasites, were the most active eukaryotes in the WWTP microbial community. Our results further showed that rDNA (abundance) and rRNA (activity) data showed profoundly different patterns, especially among the eukaryotic taxa.

### Parasite removal – predator facilitated?

Previous studies have repeatedly shown that wastewater is a hotspot of potential parasites [6, 59, 60]. These studies mostly focused on investigating the bacterial community and potentially parasitic bacteria [6], ignoring the fate of eukaryotic parasites during wastewater treatment. Our study highlights the importance of the latter group, showing a surprising diversity and abundance of gut- and feces-associated parasitic protists, particularly in the inflow (sewage). Our primer-independent findings significantly add to Maritz et al. [23], who detected various parasitic protists in raw sewage using a primer-based approach. While they identified parasitic protists such as *Blastocystis*, *Entamoeba,* and *Trichomonas*, we detected the same taxa plus numerous additional ones, including *Dientamoeba*, *Guttulinopsis*, *Giardia*, and *Rosculus*. Many of these eukaryotic parasites are known to be “long branch organisms,” i.e., organisms with highly divergent marker gene sequences that often cannot be assessed by conventional primer-based sequencing methods [49], which impedes the detection of taxa such as *Giardia* [61]. In contrast, the primer-independent shotgun data used in our study allow to assess the full spectrum of taxa. For example, Wylezich et al. [24, 25] demonstrated the use of this approach to assess the full range of eukaryotic parasites present in swine feces. It can be concluded that primer-based approaches have only limited use for monitoring eukaryotic parasites in wastewater.

An array of studies revealed that potentially harmful (parasitic) bacteria were strongly reduced during the initial phases of wastewater treatment [42, 62, 63]. Our study also shows a pronounced decrease in parasitic taxa between the inflow (sewage) and denitrification bioreactor, but, moreover, shows that this decrease also applies to eukaryotic parasites, protists in particular. This decrease can partly be explained by the transition in chemical conditions of the environment, flocculation, and sedimentation [1–3, 42]. However, our network analyses suggest that predation may also play a role, as we found parasites to co-occur with a number of other taxa within the microbial WWTP community, indicating that trophic interactions, as suggested by laboratory experiments, could be taking place [13, 14, 60, 64]. In the context of parasite removal from wastewater, the position of gut-parasitic protists is particularly interesting since many of these taxa are both predators (bacterivores) and prey. In our study, the networks of the inflow and denitrification compartments showed a high percentage of correlations (~44 %), thus putative ecological interactions, between bacteria and eukaryotes. Among these correlations, ~ 74 % involved protists, indicating their central role as bacterivorous regulators of bacterial community composition (including potentially preying on gut-parasitic bacteria). Compared to the inflow (sewage), correlations between protists and bacteria decreased in the denitrification compartment, whereas new correlations emerged between protists and their potential predators–i.e., ciliates and rotifers. Previous studies have identified ciliates (protists) and rotifers (metazoans) as potentially the most crucial predators in WWTPs [65, 66]. Our network analyses support this idea, providing evidence of trophic interactions between these predators and their protist prey, in situ.

### Contrasting abundance versus activity patterns of eukaryotic parasites: consequences for WWTP biomonitoring

Taxonomic composition of the eukaryotic fraction strongly differed between rDNA data (metagenomics) and rRNA data (metatranscriptomics) (Fig. 1). In extreme cases, we found some of the parasitic protists to be abundantly present in rDNA data but below detection level in rRNA data, or completely the other way around (Fig. 3). Such differences are generally not found in prokaryotes, where it is possible to assess “normalized activity” (RNA/DNA quotient as a measure of activity per individual) based on metagenome-assembled genomes (MAG) (see for example Herold et al. [67] and Arbas et al. [68], reporting on WWTP bacteria). Our findings show that it is not feasible to calculate this quotient for eukaryotes (especially when either rDNA or rRNA is zero); in addition, current technology does not yet allow to assess eukaryote MAGs, since eukaryotes have much larger genomes and higher variation in ribosomal gene duplication than bacteria [69].

As said, the difference in abundance (rDNA reads) versus activity (rRNA reads) was particularly strong for some of the parasitic protists (Fig. 3). In the absence of their natural hosts, we expected these parasites to become dormant, i.e., low in activity and potentially forming resting stages [70]. This was indeed found for the taxa *Blastocystis*, *Copromyxa*, and *Guttulinopsis*, whose presence could be detected via rDNA but whose activity was so low that it mostly fell below the sensitivity threshold of our rRNA sequencing (Fig. 3). In contrast, the taxa *Dientamoeba*, *Entamoeba*, *Giardia*, and *Rosculus* were hardly present in the rDNA data but showed a high expression of ribosomal genes, indicating high activity and even potential reproduction [69]. Outstanding was the high proportion of *Rosculus* in rRNA data, making up to ~ 84 % of the eukaryotic fraction. *Rosculus* is known to be highly abundant and active in feces [49], and, as this study indicates, also in sewage within WWTPs.

The importance and ecological meaning of these differences in abundance versus activity data were further revealed in our network analysis (Fig. 4). As expected, the activity-based rRNA networks showed a higher number of edges, i.e., putative interactions, than the rDNA-based networks, because rRNA data reflect the active part of the community. More importantly, the rRNA-based networks revealed associations between parasitic protists and their potential predators (ciliates and rotifers) that were not detected in the rDNA networks. Thus, the very low abundance (rDNA reads) of the parasitic protists *Dientamoeba*, *Entamoeba*, *Giardia*, and *Rosculus* may be explained by predation. At the same time, their observed high activity (rRNA reads) and strong network correlations with bacteria suggest that these protists, while being preyed upon, themselves were actively feeding on bacteria (Fig. 4).

## Conclusions

Our results are of particular interest for biomonitoring to evaluate wastewater treatment efficiency [22, 63, 71]. Especially in developing countries, the treatment of wastewater may be insufficient before re-introduction into the water system or re-use for agricultural purposes [6, 17, 59, 72]. According to Cai et al. [42], this applies, for instance, to around 80 % of sewage in India. Subsequently, potentially infectious and harmful parasitic microorganisms become widespread and form a threat to public health when present in drinking water, water recreation areas, and aquatic food production systems [42, 73–75]. Our results clearly show that biomonitoring of wastewater treatment efficiency via molecular methods (“-omics”) can be greatly improved by using primer-independent shotgun approaches to ensure adequate detection of parasitic protists. Combining shotgun metagenomics with shotgun metatranscriptomics allows to monitor both abundance and activity of this important group of microeukaryotic parasites. This improvement is crucial for reducing the public health risks associated with insufficiently treated wastewater.

## Supplementary Information

**Additional file 1: Supplementary Figure 1.** Comparing microbial communities between WWTP locations to identify outliers. Graphs showing multivariate dispersion (A) and beta diversity (B) of microbial community composition at N=11 WWTP locations, based on metagenomic data (left-hand side) and metatranscriptomic data (right-hand side). For multivariate dispersion (A), NMDS plots were calculated based on Bray-Curtis dissimilarities. Lines (color-coded by location) link samples at each location to their centromere. For beta diversity (B), boxplots show the 25 % and 75 % percentiles and medians of Bray-Curtis dissimilarities. Points are color and symbol-coded by WWTP compartments: INF = inflow (sewage), DNF = denitrification bioreactor, NFC = nitrification bioreactor, EFF = effluent (treated water). In beta diversity based on metatranscriptomic data (lower row, right-hand side), significant differences between location “FD” and the other locations are indicated with asterisks (unpaired two-sample Wilcoxon test, * *p* < 0.05; ** *p* < 0.01; *** *p* < 0.001). Based on these results, location FD was excluded from further analysis. **Supplementary Figure 2.** Assessment of the variation caused by sampling processing (sequencing). NMDS plot based on Bray-Curtis dissimilarities derived from metatranscriptomic data, comparing microbial community composition across WWTP compartments and locations. The variation caused by sample processing is shown for one location, “ZR”, showing three sequencing replicates from the inflow (INF) (replicates indicated by yellow asterisks). Based on this comparison, we concluded that variation caused by sequencing was low. Compartments: INF = inflow (sewage), DNF = denitrification bioreactor, NFC = nitrification bioreactor, EFF = effluent (treated water). **Supplementary Figure 3.** Rarefaction curves for metagenomic (rDNA) and metatranscriptomic (rRNA) data. Curves showing the number of reads as a function of the number of OTUs identified (N=37 samples, i.e. one sample from each WWTP compartment (4) at each WWTP location (10), excluding 3 samples because of exceptionally low sequencing-depth). Samples are color-coded by compartment: INF = inflow (sewage), DNF = denitrification bioreactor, NFC = nitrification bioreactor, EFF = effluent (treated water). **Supplementary Figure 4.** Total number of rDNA and rRNA sequences. Boxplots showing the 25 % and 75 % percentiles and medians of the total number of rDNA (metagenomics) and rRNA (metatranscriptomics) sequences for (A) the total community and (B) the parasitic community, comparing prokaryotes (blue) and eukaryotes (yellow). Compartments: INF = inflow (sewage), DNF = denitrification bioreactor, NFC = nitrification bioreactor, EFF = effluent (treated water). **Supplementary Figure 5.** Microbial community structure and environmental factors across WWTPs. NMDS biplots based on Bray-Curtis dissimilarities showing microbial community composition across WWTP compartments and locations, in association with environmental data. Metagenomic and metatranscriptomic data are shown separately. Samples are color-coded and grouped (ellipses) by compartment. Significant environmental vectors are shown as arrows (* *p* < 0.05; ** *p* < 0.01; *** *p* < 0.001). Compartments: INF = inflow (sewage), DNF = denitrification bioreactor, NFC = nitrification bioreactor, EFF = effluent (treated water). Environmental vectors: DO = dissolved oxygen, DOC = dissolved organic carbon, HRT = Hydraulic retention time, TN = total nitrogen, TP = total phosphorus. **Supplementary Figure 6.** Microbial community structure and environmental factors in the separate WWTP compartments. NMDS biplots based on Bray-Curtis dissimilarities, showing microbial community composition in association with environmental data for each WWTP compartment. Metagenomic and metatranscriptomic data are shown separately. The distribution of the samples (symbol-coded by WWTP location) is visualized by the ellipses. Significant environmental vectors are shown as arrows. Compartments: INF = inflow (sewage), DNF = denitrification bioreactor, NFC = nitrification bioreactor, EFF = effluent (treated water). Environmental vectors: TN = total nitrogen. **Supplementary Figure 7.** Co-occurrence networks of parasitic orders in the four WWTP compartments. Networks showing correlations derived from co-occurrence network inferences for each WWTP compartment, based on metagenomic (first row) and metatranscriptomic (second row) data. Only associations that involve parasites are shown. Nodes represent genera grouped at the order level and trait level (red nodes: parasitic taxa; yellow nodes: free-living taxa), with node size proportional to the total number of reads for each order. Edges represent correlations between taxa (blue lines: positive correlations; red lines: negative correlations), with line thickness proportional to the number of genera per order involved. Compartments: INF = inflow (sewage), DNF = denitrification bioreactor, NFC = nitrification bioreactor, EFF = effluent. Abbreviations for Archaea: E = Euryarchaeota, O = Others. Abbreviations for bacteria: A = Actinobacteria, B = Bacteroidetes, C = Chloroflexi, F = Firmicutes, O = Others, Pl = Planctomycetes, Pr = Proteobacteria, T = Tenericutes, V = Verrucomicrobia. Abbreviations for Proteobacteria: Alpha = Alphaproteobacteria, Beta = Betaproteobacteria, Gamma = Gammaproteobacteria, Delta = Deltaproteobacteria, O = Others. Abbreviations Fungi: Ascomycota = A, Basidiomycota = B, Others = O. Abbreviations for protists: Ce = Cercozoa (*including *Rosculus*), Ci = Ciliophora, Co = Conosa, Db = Discoba, Di = Discosea, Ms = Mesomycetozoa, Mt = Metamonada, O = Others, S = Stramenopiles, T = Tubulinea. **Supplementary Table 1.** Microbial community composition after quality filtering. Total number and relative number (%) of ribosomal reads and OTUs in the metagenomic (rDNA) and metatranscriptomic (rRNA) data of 10 WWTP locations, for prokaryotes (bacteria and Archaea) and eukaryotes (protists, fungi and microscopic metazoa). **Supplementary Table 2.** Parasitic genera in WWTPs based on both metagenomic and metatranscriptomic data. Overview of all parasitic genera identified in the WWTP samples. **Supplementary Table 3.** Comparing the total number of rDNA and rRNA sequences. Pair-wise comparison of the total number of eukaryotic and prokaryotic sequences for the total community and the parasitic community, contrasting the inflow (INF) with the denitrification bioreactor (DNF), the denitrification bioreactor (DNF) with the nitrification bioreactor (NFC), and the nitrification bioreactor (NFC) with the effluent (EFF). Sign test (* *p* < 0.05; ** *p* < 0.01; *** *p* < 0.001). **Supplementary Table 4.** Comparing the abundance of parasitic protists between WWTP compartments. Pair-wise comparison of relative abundances of parasitic protist taxa in metagenomic and metatranscriptomic data, contrasting the inflow (INF) with the denitrification bioreactor (DNF), and the nitrification bioreactor (NFC) with the effluent (EFF). Sign test (* *p* < 0.05; ** *p* < 0.01; *** *p* < 0.001). **Supplementary Table 5.** Overview of the most numerous orders in WWTPs. Overview of the most numerous orders shown in Fig. 1. Numbers show their mean relative abundances across all compartments and locations (total N=40 samples) for both rDNA and rRNA data, as well as the absolute difference between these relative DNA and RNA abundances, per order.

## Data Availability

The analyzed data was submitted to the European Nucleotide Archive under the accession numbers PRJEB28815, PRJEB28830, PRJEB30264, PRJEB28728, PRJEB28831, and PRJEB28737. Tables with metadata are included in the publication Ju et al. [28]. The code for the presented analyses are available over GitHub under the following link: https://github.com/JFreude/MicroeukaryoticGutParasitesInWastewaterTreatmentPlants

## Abbreviations

WWTPs: Wastewater treatment plants
rDNA: Ribosomal DNA
rRNA: Ribosomal RNA
INF: Sewage-inflow
DNF: Denitrification bioreactor
NFC: Nitrification bioreactor
EFF: Effluent
OTUs: Operational taxonomic units
MAG: Metagenome-assembled genomes
