# Supplementary material for: Microeukaryotic gut parasites in wastewater treatment plants: diversity, activity, and removal
Source: Microbiome. 2022 Feb 9;10:27. doi: 10.1186/s40168-022-01225-y (PMC8827150; doi:10.1186/s40168-022-01225-y)
Supplement: Supplementary file 2 — Additional file 1: Supplementary Figure 1. Comparing microbial communities between WWTP locations to identify outliers. Graphs showing multivariate dispersion (A) and beta diversity (B) of microbial community composition at N=11 WWTP locations, based on metagenomic data (left-hand side) and metatranscriptomic data (right-hand side). For multivariate dispersion (A), NMDS plots were calculated based on Bray-Curtis dissimilarities. Lines (color-coded by location) link samples at each location to their centromere. For beta diversity (B), boxplots show the 25 % and 75 % percentiles and medians of Bray-Curtis dissimilarities. Points are color and symbol-coded by WWTP compartments: INF = inflow (sewage), DNF = denitrification bioreactor, NFC = nitrification bioreactor, EFF = effluent (treated water). In beta diversity based on metatranscriptomic data (lower row, right-hand side), significant differences between location “FD” and the other locations are indicated with asterisks (unpaired two-sample Wilcoxon test, * p < 0.05; ** p < 0.01; *** p < 0.001). Based on these results, location FD was excluded from further analysis. Supplementary Figure 2. Assessment of the variation caused by sampling processing (sequencing). NMDS plot based on Bray-Curtis dissimilarities derived from metatranscriptomic data, comparing microbial community composition across WWTP compartments and locations. The variation caused by sample processing is shown for one location, “ZR”, showing three sequencing replicates from the inflow (INF) (replicates indicated by yellow asterisks). Based on this comparison, we concluded that variation caused by sequencing was low. Compartments: INF = inflow (sewage), DNF = denitrification bioreactor, NFC = nitrification bioreactor, EFF = effluent (treated water). Supplementary Figure 3. Rarefaction curves for metagenomic (rDNA) and metatranscriptomic (rRNA) data. Curves showing the number of reads as a function of the number of OTUs identified (N=37 samples, i.e. one [file 40168_2022_1225_MOESM2_ESM.pdf]

Supplementary information

Microeukaryotic gut parasites in wastewater treatment plants: Diversity, activity and removal

Authors Jule Freudenthal[a], Feng Ju[b, c], Helmut Bürgmann[d], and Kenneth Dumack[a]

Corresponding author Kenneth Dumack

Phone: +49-(0)221-470-6635 Fax: +49-(0)221-470-5038

[a] University of Cologne, Terrestrial Ecology, Institute of Zoology, Zülpicher Str. 47b, 50674 Köln, Germany

[b] Key Laboratory of Coastal Environment and Resources of Zhejiang Province, School of Engineering, Westlake University, 310024 Hangzhou, China

[c] Institute of Advanced Technology, Westlake Institute for Advanced Study, 310024 Hangzhou, China.

[d] Eawag, Swiss Federal Institute of Aquatic Science and Technology, 6047 Kastanienbaum, Switzerland

jule.freudenthal@uni-koeln.de, jufeng@westlake.edu.cn, helmut.buergmann@eawag.ch, kenneth.dumack@uni-koeln.de

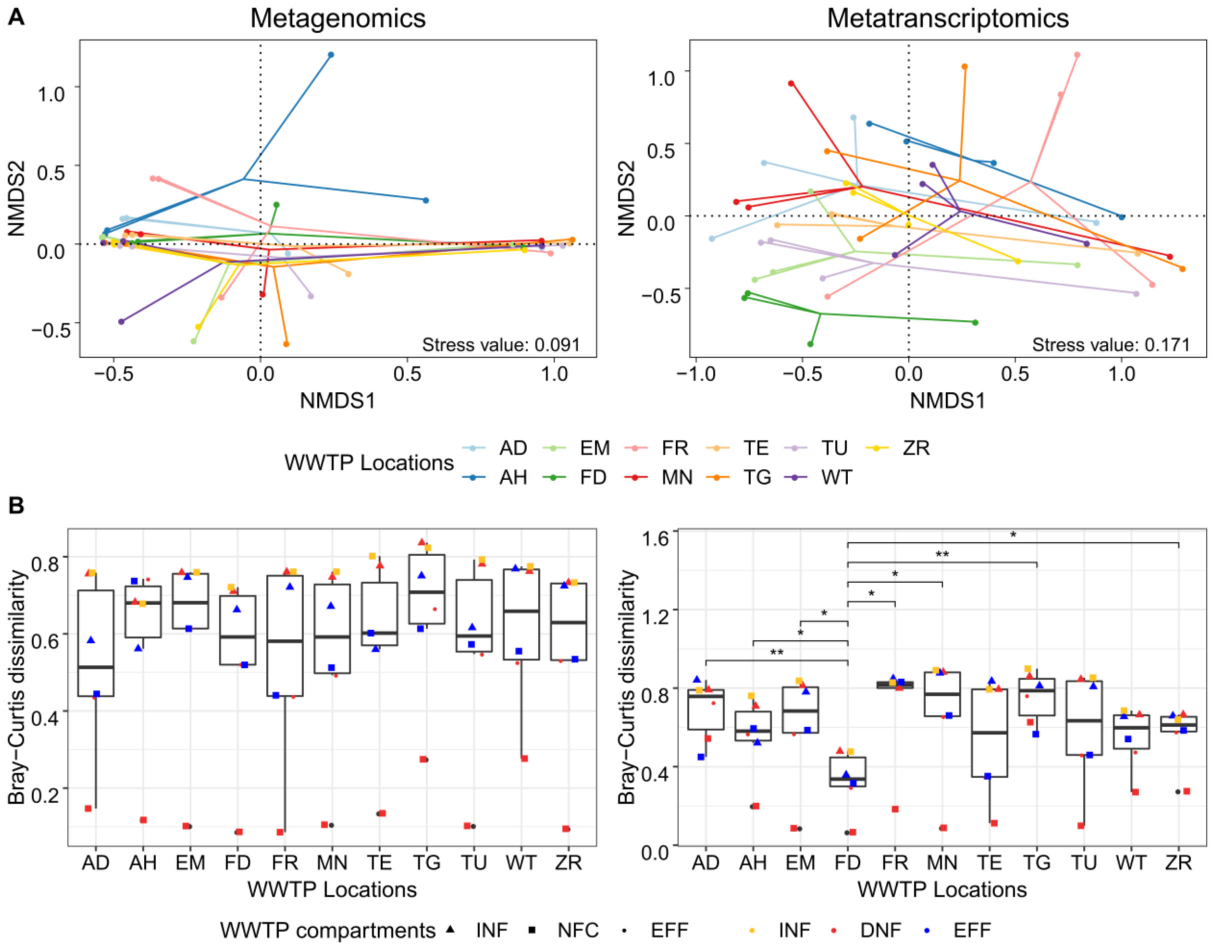

Supplementary Figure 1: Comparing microbial communities between WWTP locations to identify outliers. Graphs showing multivariate dispersion **(A)** and beta diversity **(B)** of microbial community composition at N=11 WWTP locations, based on metagenomic data (left-hand side) and metatranscriptomic data (right-hand side). For multivariate dispersion **(A)**, NMDS plots were calculated based on Bray-Curtis dissimilarities. Lines (color-coded by location) link samples at each location to their centromere. For beta diversity **(B)**, boxplots show the 25 % and 75 % percentiles and medians of Bray-Curtis dissimilarities. Points are color and symbol-coded by WWTP compartments: INF = inflow (sewage), DNF = denitrification bioreactor, NFC = nitrification bioreactor, EFF = effluent (treated water). In beta diversity based on metatranscriptomic data (lower row, right-hand side), significant differences between location "FD" and the other locations are indicated with asterisks (unpaired two-sample Wilcoxon test, * $p < 0.05$; ** $p < 0.01$; *** $p < 0.001$). **Based on these results, location FD was excluded from further analysis.**

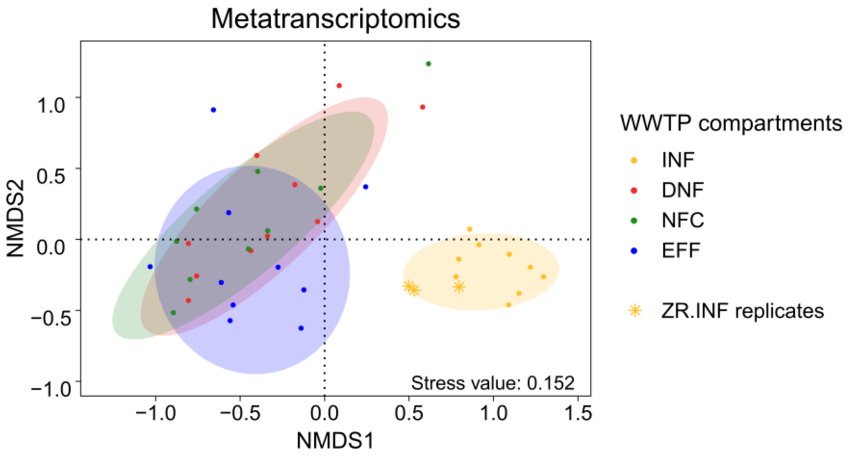

Supplementary Figure 2: Assessment of the variation caused by sampling processing (sequencing). NMDS plot based on Bray-Curtis dissimilarities derived from metatranscriptomic data, comparing microbial community composition across WWTP compartments and locations. The variation caused by sample processing is shown for one location, "ZR", showing three sequencing replicates from the inflow (INF) (replicates indicated by yellow asterisks). **Based on this comparison, we concluded that variation caused by sequencing was low.** Compartments: INF = inflow (sewage), DNF = denitrification bioreactor, NFC = nitrification bioreactor, EFF = effluent (treated water).

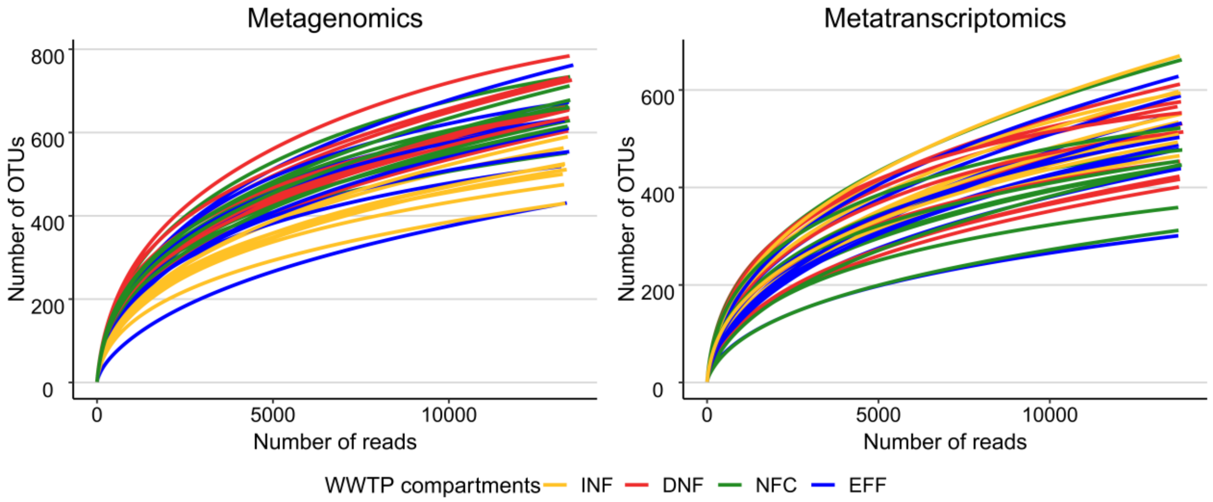

Supplementary Figure 3: Rarefaction curves for metagenomic (rDNA) and metatranscriptomic (rRNA) data. Curves showing the number of reads as a function of the number of OTUs identified (N=37 samples, i.e. one sample from each WWTP compartment (4) at each WWTP location (10), excluding 3 samples because of exceptionally low sequencing-depth). Samples are color-coded by compartment: INF = inflow (sewage), DNF = denitrification bioreactor, NFC = nitrification bioreactor, EFF = effluent (treated water).

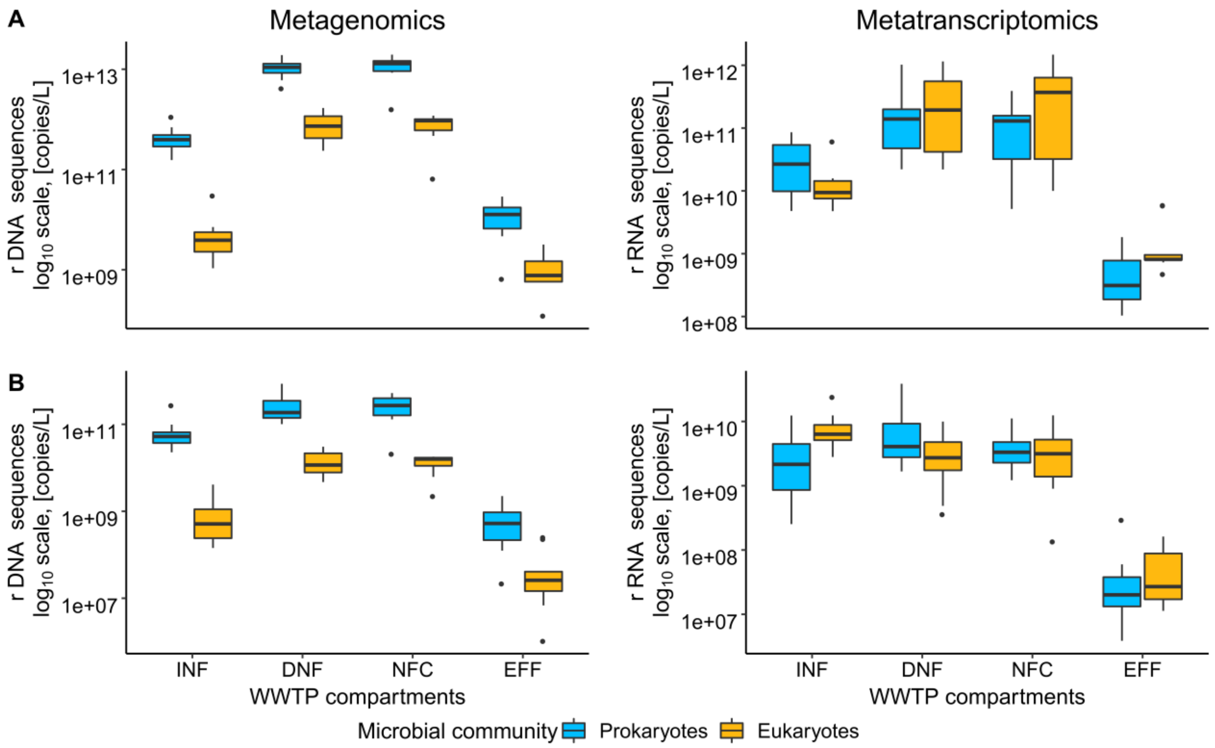

Supplementary Figure 4: Total number of rDNA and rRNA sequences. Boxplots showing the 25% and 75% percentiles and medians of the total number of rDNA (metagenomics) and rRNA (metatranscriptomics) sequences for **(A)** the total community and **(B)** the parasitic community, comparing prokaryotes (blue) and eukaryotes (yellow). Compartments: INF = inflow (sewage), DNF = denitrification bioreactor, NFC = nitrification bioreactor, EFF = effluent (treated water).

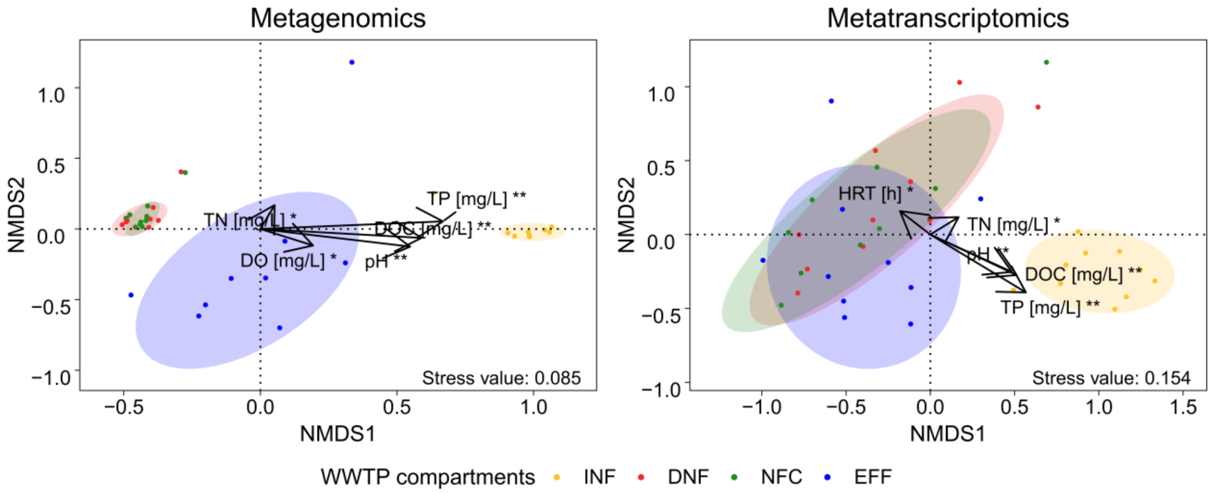

Supplementary Figure 5: Microbial community structure and environmental factors across WWTPs. NMDS biplots based on Bray-Curtis dissimilarities showing microbial community composition across WWTP compartments and locations, in association with environmental data. Metagenomic and metatranscriptomic data are shown separately. Samples are color-coded and grouped (ellipses) by compartment. Significant environmental vectors are shown as arrows (* $p < 0.05$; ** $p < 0.01$; *** $p < 0.001$). Compartments: INF = inflow (sewage), DNF = denitrification bioreactor, NFC = nitrification bioreactor, EFF = effluent (treated water). Environmental vectors: DO = dissolved oxygen, DOC = dissolved organic carbon, HRT = Hydraulic retention time, TN = total nitrogen, TP = total phosphorus.

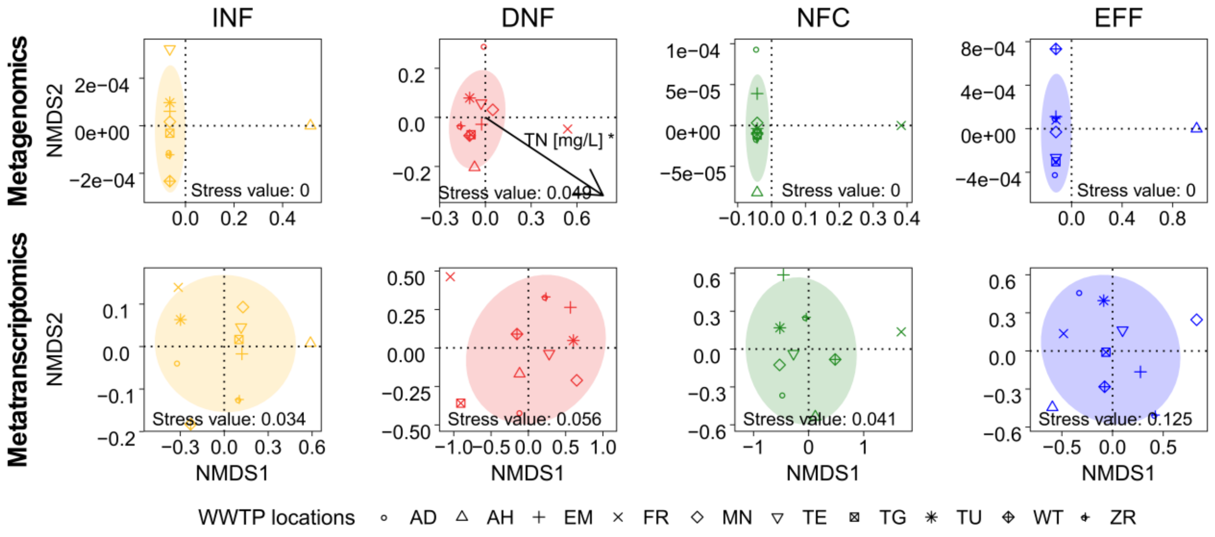

Supplementary Figure 6: Microbial community structure and environmental factors in the separate WWTP compartments. NMDS biplots based on Bray-Curtis dissimilarities, showing microbial community composition in association with environmental data for each WWTP compartment. Metagenomic and metatranscriptomic data are shown separately. The distribution of the samples (symbol-coded by WWTP location) is visualized by the ellipses. Significant environmental vectors are shown as arrows. Compartments: INF = inflow (sewage), DNF = denitrification bioreactor, NFC = nitrification bioreactor, EFF = effluent (treated water). Environmental vectors: TN = total nitrogen.

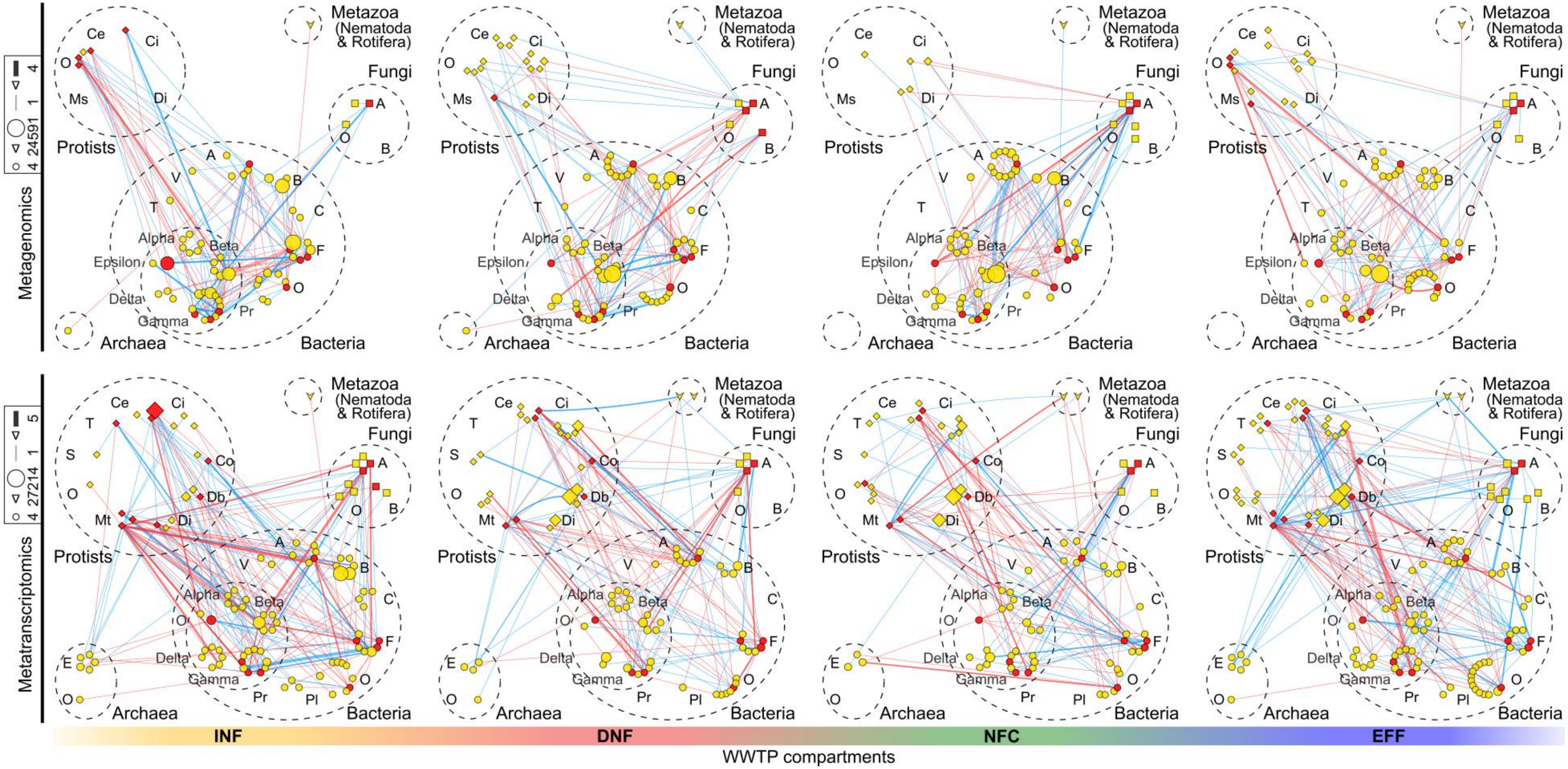

Supplementary Figure 7: Co-occurrence networks of parasitic orders in the four WWTP compartments. Networks showing correlations derived from co-occurrence network inferences for each WWTP compartment, based on metagenomic (first row) and metatranscriptomic (second row) data. Only associations that involve parasites are shown. Nodes represent genera grouped at the order level and trait level (red nodes: parasitic taxa; yellow nodes: free-living taxa), with node size proportional to the total number of reads for each order. Edges represent correlations between taxa (blue lines: positive correlations; red lines: negative correlations), with line thickness proportional to the number of genera per order involved. Compartments: INF = inflow (sewage), DNF = denitrification bioreactor, NFC = nitrification bioreactor, EFF = effluent. Abbreviations for Archaea: E = Euryarchaeota, O = Others. Abbreviations for bacteria: A = Actinobacteria, B = Bacteroidetes, C = Chloroflexi, F = Firmicutes, O = Others, Pl = Planctomycetes, Pr = Proteobacteria, T = Tenericutes, V = Verrucomicrobia. Abbreviations for Proteobacteria: Alpha = Alphaproteobacteria, Beta = Betaproteobacteria, Gamma = Gammaproteobacteria, Delta = Deltaproteobacteria, O = Others. Abbreviations Fungi: Ascomycota = A, Basidiomycota = B, Others = O. Abbreviations for protists: Ce = Cercozoa (*including *Rosculus*), Ci = Ciliophora, Co = Conosa, Db = Discoba, Di = Discosea, Ms = Mesomycetozoa, Mt = Metamonada, O = Others, S = Stramenopiles, T = Tubulinea.

Supplementary Table 1: Microbial community composition after quality filtering. Total number and relative number (%) of ribosomal reads and OTUs in the metagenomic (rDNA) and metatranscriptomic (rRNA) data of 10 WWTP locations, for prokaryotes (bacteria and Archaea) and eukaryotes (protists, fungi and microscopic metazoa).

		Metagenomics				Metatranscriptomics			
		Number of reads	[%]	Number of OTUs	[%]	Number of reads	[%]	Number of OTUs	[%]
	Prokaryotes	479487	~ 94.34	1366	~ 70.16	228569	~ 42.49	1142	~ 60.52
Eukaryotes	Protists	23198	~ 4.56	388	~ 19.93	294510	~ 54.75	480	~ 25.44
Eukaryotes	Fungi	3763	~ 0.74	126	~ 6.47	9980	~ 1.86	196	~ 10.39
Eukaryotes	Metazoa	1802	~ 0.35	67	~ 3.44	4876	~ 0.91	69	~ 3.66

Supplementary Table 2: Parasitic genera in WWTPs based on both metagenomic and metatranscriptomic data. Overview of all parasitic genera identified in the WWTP samples.

Genera	Microbial community	Genera	Microbial community
Arcobacter	Prokaryotes	*Crithidia*	Protists
Bacillus	Prokaryotes	*Cryptosporidium*	Protists
Campylobacter	Prokaryotes	*Dientamoeba*	Protists
Clostridium	Prokaryotes	*Entamoeba*	Protists
Corynebacterium	Prokaryotes	*Enterobryus*	Protists
Enterococcus	Prokaryotes	*Enteromonas*	Protists
Helicobacter	Prokaryotes	*Giardia*	Protists
Klebsiella	Prokaryotes	Gregarines_XX	Protists
Legionella	Prokaryotes	*Guttulinopsis*	Protists
Leptospira	Prokaryotes	*Helkesimastix*	Protists
Listeria	Prokaryotes	*Herpetomonas*	Protists
Mycobacterium	Prokaryotes	*Hexamita*	Protists
Pseudomonas	Prokaryotes	Hexamitinae-Enteromonadida_X	Protists
Salmonella	Prokaryotes	*Ichthyophonus*	Protists
Vibrio	Prokaryotes	*Lacusteria*	Protists
Yersinia	Prokaryotes	*Leishmania*	Protists
Acremonium	Fungi	*Leptomonas*	Protists
Aspergillus	Fungi	*Monocystis*	Protists
Candida	Fungi	*Paratrypanosoma*	Protists
Cladophialophora	Fungi	Perkinsida_XXX	Protists
Cryptococcus	Fungi	*Phytomonas*	Protists
Fusarium	Fungi	*Pseudotrichomonas*	Protists
Mucor	Fungi	*Rhinosporidium*	Protists
Ochroconis	Fungi	Rhynosporidae_X	Protists
Penicillium	Fungi	*Rosculus*	Protists
Rhodotorula	Fungi	*Sainouron*	Protists
Trichosporon	Fungi	*Sappinia*	Protists
Ascaris	Metazoa	*Sphaerothecum*	Protists
Acanthamoeba	Protists	*Tetratrichomonas*	Protists
Anurofeca	Protists	*Trepomonas*	Protists
Blastocystis	Protists	*Trichomitus*	Protists
Blastodinium	Protists	Trichomonadidae_X	Protists
Blechomonas	Protists	*Trichomonas*	Protists
Copromyxa	Protists	*Trimitus*	Protists
Creolimax	Protists	Trypanosomatidae_X	Protists

Supplementary Table 3: Comparing the total number of rDNA and rRNA sequences. Pair-wise comparison of the total number of eukaryotic and prokaryotic sequences for the total community and the parasitic community, contrasting the inflow (INF) with the denitrification bioreactor (DNF), the denitrification bioreactor (DNF) with the nitrification bioreactor (NFC), and the nitrification bioreactor (NFC) with the effluent (EFF). Sign test (* $p < 0.05$; ** $p < 0.01$; *** $p < 0.001$).

Data	Microbial commuity	Data subset	Group 1	Group 2	p-value	
Metagenomics	Eukaryotes	All	INF	DNF	0.00195	**
Metagenomics	Eukaryotes	All	DNF	NFC	1.00000	
Metagenomics	Eukaryotes	All	NFC	EFF	0.00195	**
Metagenomics	Eukaryotes	Parasites	INF	DNF	0.00195	**
Metagenomics	Eukaryotes	Parasites	DNF	NFC	0.75400	
Metagenomics	Eukaryotes	Parasites	NFC	EFF	0.00195	**
Metagenomics	Prokaryotes	All	INF	DNF	0.00195	**
Metagenomics	Prokaryotes	All	DNF	NFC	1.00000	
Metagenomics	Prokaryotes	All	NFC	EFF	0.00195	**
Metagenomics	Prokaryotes	Parasites	INF	DNF	0.00195	**
Metagenomics	Prokaryotes	Parasites	DNF	NFC	1.00000	
Metagenomics	Prokaryotes	Parasites	NFC	EFF	0.00195	**
Metatranscriptomics	Eukaryotes	All	INF	DNF	0.00195	**
Metatranscriptomics	Eukaryotes	All	DNF	NFC	1.00000	
Metatranscriptomics	Eukaryotes	All	NFC	EFF	0.00195	**
Metatranscriptomics	Eukaryotes	Parasites	INF	DNF	0.10900	
Metatranscriptomics	Eukaryotes	Parasites	DNF	NFC	0.75400	
Metatranscriptomics	Eukaryotes	Parasites	NFC	EFF	0.00195	**
Metatranscriptomics	Prokaryotes	All	INF	DNF	0.02150	*
Metatranscriptomics	Prokaryotes	All	DNF	NFC	1.00000	
Metatranscriptomics	Prokaryotes	All	NFC	EFF	0.00195	**
Metatranscriptomics	Prokaryotes	Parasites	INF	DNF	0.10900	
Metatranscriptomics	Prokaryotes	Parasites	DNF	NFC	1.00000	
Metatranscriptomics	Prokaryotes	Parasites	NFC	EFF	0.00195	**

Supplementary Table 4: Comparing the abundance of parasitic protists between WWTP compartments. Pair-wise comparison of relative abundances of parasitic protist taxa in metagenomic and metatranscriptomic data, contrasting the inflow (INF) with the denitrification bioreactor (DNF), and the nitrification bioreactor (NFC) with the effluent (EFF). Sign test (* p < 0.05; ** p < 0.01; *** p < 0.001).

Data	Genera	Group 1	Group 2	p-value	
Metagenomics	*Blastocystis*	DNF	INF	0.00781	**
Metagenomics	*Copromyxa*	DNF	INF	0.0625	
Metagenomics	*Dientamoeba*	DNF	INF	0.5	
Metagenomics	*Entamoeba*	DNF	INF	1	
Metagenomics	*Giardia*	DNF	INF	0.625	
Metagenomics	*Guttulinopsis*	DNF	INF	0.25	
Metagenomics	*Rosculus*	DNF	INF	0.00391	**
Metatranscriptomics	*Blastocystis*	DNF	INF	0.625	
Metatranscriptomics	*Copromyxa*	DNF	INF	0.00781	**
Metatranscriptomics	*Dientamoeba*	DNF	INF	0.00195	**
Metatranscriptomics	*Entamoeba*	DNF	INF	0.00195	**
Metatranscriptomics	*Giardia*	DNF	INF	0.0215	*
Metatranscriptomics	*Guttulinopsis*	DNF	INF	0.00195	**
Metatranscriptomics	*Rosculus*	DNF	INF	0.00195	**
Metagenomics	*Blastocystis*	EFF	NFC	0.687	
Metagenomics	*Copromyxa*	EFF	NFC	1	
Metagenomics	*Dientamoeba*	EFF	NFC	1	
Metagenomics	*Entamoeba*	EFF	NFC	1	
Metagenomics	*Giardia*	EFF	NFC	1	
Metagenomics	*Guttulinopsis*	EFF	NFC	0.5	
Metagenomics	*Rosculus*	EFF	NFC	0.25	
Metatranscriptomics	*Blastocystis*	EFF	NFC	1	
Metatranscriptomics	*Copromyxa*	EFF	NFC	0.0313	*
Metatranscriptomics	*Dientamoeba*	EFF	NFC	1	
Metatranscriptomics	*Entamoeba*	EFF	NFC	1	
Metatranscriptomics	*Giardia*	EFF	NFC	0.18	
Metatranscriptomics	*Guttulinopsis*	EFF	NFC	0.508	
Metatranscriptomics	*Rosculus*	EFF	NFC	0.0391	*

Supplementary Table 5: Overview of the most numerous orders in WWTPs. Overview of the most numerous orders shown in Fig. 1. Numbers show their mean relative abundances across all compartments and locations (total N=40 samples) for both rDNA and rRNA data, as well as the absolute difference between these relative DNA and RNA abundances, per order.

	Microbial community		Order	DNA [%]	RNA [%]	Absolute difference [%]
	Prokaryotes		Acidimicrobiales	2.36		
	Prokaryotes		Bacteroidales	4.46	6.78	2.32
	Prokaryotes		Flavobacteriales	3.82	7.19	3.37
	Prokaryotes		Sphingobacteriales	6.82	7.08	0.26
	Prokaryotes		Bacillales		3.51	
	Prokaryotes		Clostridiales	6.14	3.91	2.23
	Prokaryotes		Parcubacteria_XX	2.96	3.42	0.46
	Prokaryotes		Burkholderiales	17.98	12.11	5.87
	Prokaryotes		Rhodocyclales	9.1	4.06	5.05
	Prokaryotes		Enterobacteriales		4.05	
	Prokaryotes	Proteobacteria	Pseudomonadales	5.4	4.48	0.92
	Prokaryotes		Myxococcales	3.3	6.45	3.15
	Prokaryotes		Campylobacterales	4.17		
	Prokaryotes		Others	33.49	36.96	3.47
	Protists		Cryomonadida	7.08		
	Protists		Imbricatea_X		1.29	
	Protists		Sainouridea		15.43	
	Protists		Cyrtophoria	3.77	1.57	2.2
	Protists		Euplotia	4.12	1.68	2.44
	Protists		Haptoria	3.43		
	Protists		Peritrichia	26.41	10.73	15.68
Eukaryotes	Protists		Suctoria	4.16		
	Protists		Euglenida		13.77	
	Protists		Heterolobosea_X		4.13	
	Protists		Kinetoplastida	3.75	23.67	19.92
	Protists		Himatismenida		1.57	
	Protists		Vannellida	2.18	12.86	10.68
	Fungi		Pezizomycotina	7.11	2.79	4.32
	Fungi		Saccharomycotina	5.53		
	Metazoa		Rotifera_X	2.91		
	Eukaryotes		Others	29.56	10.52	19.04
